# Supplementary material for: Identification of photoperiod-induced specific miRNAs in the adrenal glands of Sunite sheep (Ovis aries)
Source: Front Vet Sci. 2022 Jul 22;9:888207. doi: 10.3389/fvets.2022.888207 (PMC9354845; doi:10.3389/fvets.2022.888207)
Supplement: Supplementary file 1 [file Data_Sheet_1.ZIP › Supplementary file/miRDeep2 file.zip!/miRDeep2 file/Know_Structure/SP42A3/oar-mir-494.pdf]

miRBase precursor : oar-mir-494
Total read count : 17639
oar-miR-494-5p read count : 20
oar-miR-494-3p read count : 17618
remaining reads : 1

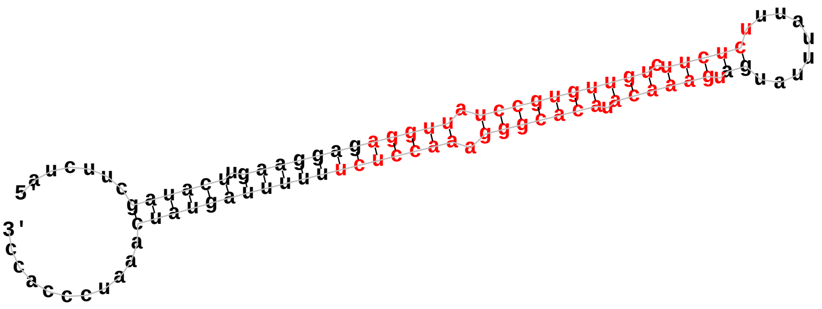

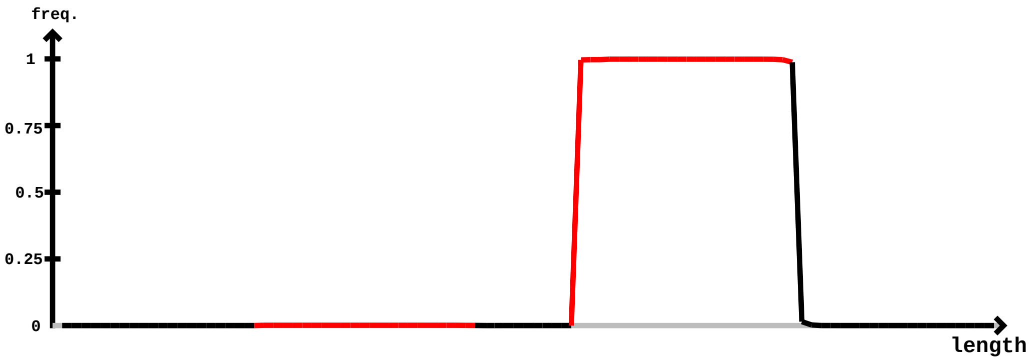

oar-miR-494-3p
oar-miR-494-5p

```
5'- aucuucgauacuugaaggagagguuauccguguugucuucucuuuauuuaugaugaaacauacacgggaaaccucuuuuuuaguaucaaaucccacc   -3'    exp
    ......((((((.((((((((((((.((((((((((.(((((.........)).)))))).)))))).))))))))))))))))))))..........    reads     mm      sample
    ....................agguuauccguguugucuucu........................................................    8         0       seq
    ....................agguuauccguguugucuucuc.......................................................    4         0       seq
    ....................agguuauccguguugucuucucu......................................................    8         0       seq
    .............................................auuuaugaugaGacaua...................................    1         1       seq
    .....................................................ugaaacauacacgggaaac.........................    1         0       seq
    .....................................................ugaaacauacacgggaaacc........................    5         0       seq
    .....................................................ugaaacauacacgggaaaccu.......................    29        0       seq
    .....................................................ugaaacauacacgggaaaccuc......................    167       0       seq
    .....................................................ugaaacauacacgggaaaccucu.....................    17138     0       seq
    .....................................................ugaaacauacacgggaaaccucuu....................    215       0       seq
    .....................................................ugaaacauacacgggaaaccucuuu...................    15        0       seq
    ......................................................gaaacauacacgggaaaccucu.....................    16        0       seq
    .......................................................aaacauacacgggaaaccucu.....................    3         0       seq
    ........................................................aacauacacgggaaaccucuu....................    2         0       seq
    ........................................................aacauacacgggaaaccucuuu...................    26        0       seq
    ........................................................aacauacacgggaaaccucuuuuu.................    1         0       seq
```
